# ANGPTL4 variants E40K and T266M are associated with lower fasting triglyceride levels and predicts cardiovascular disease risk in Type 2 diabetic Tunisian population

**DOI:** 10.1186/s12944-016-0231-6

**Published:** 2016-03-23

**Authors:** Kaouthar Abid, Thouraya Trimeche, Donia Mili, Mohamed Amine Msolli, Imen Trabelsi, Semir Nouira, Abderraouf Kenani

**Affiliations:** Laboratory of biochemistry, UR 12ES08, Faculty of Medicine, 5000 Monastir, Tunisia; Emergency Medicine, F. Bourguiba University Hospital, Monastir, Tunisia; Research Unit: UR 12ES09 Dyslipidemia and Atherogenesis, Faculty of Medicine, Monastir, 5000 Tunisia

**Keywords:** Angiopoietin-like protein 4, CVD risk factors, Coronary artery disease, Metabolic syndrome, Obesity, TG levels

## Abstract

**Background:**

Angiopoietin-like protein 4 (ANGPTL4) is a metabolic factor that increases plasma triglyceride levels by inhibiting lipoprotein lipase (LPL). The objective of this study was to investigate the association of ANGPTL4 variants (E40K and T266M) with triglyceride levels and with cardiovascular risk factors, such as metabolic syndrome (MetS) and obesity in type 2 diabetic Tunisian population.

**Methods:**

We investigated the effect of the tagging single nucleotide polymorphisms (SNPs) rs1044250 (T266M) and rs116843064 (E40K) with triglyceride (TG) levels and CAD risk factors in a cohort of 220 patients undergoing coronary angiography for the evaluation of stable CAD, all of whom had (type 2 diabetes) T2D and were at least overweight. Multivariate logistic regressions were performed on association studies.

**Results:**

TT genotype of rs1044250 (T266M variant) showed a protective effect on CVD risk in CAD group patients (OR 1.92, 95 % CI 0.601.42, *p* =0.05) compared with control Group patients (OR 1.17, 95 % CI 0.70–1.66, *p* = 0.72). Likewise, GA genotype of rs116843064 (E40K variant): (OR 0.74, 95 % CI 0.54–1.65, *p* =0.01) for the CAD group compared with control Group patients (OR 1.12, 95 % CI 0.68–1.74, *p* = 0.074).

**Conclusions:**

ANGPTL4 variants are associated with, not only lower fasting triglyceride levels, but also a decreased cardiovascular risk in T2D Tunisian patients. So, T266M and E40K polymorphism predicts cardiovascular disease risk in Type 2 diabetic Tunisian population

## Background

The angiopoietin-like protein 4 (ANGPTL4) peptide, is a well established regulator of LPL activity and triglyceride levels. In vitro studies confirm that Angptl4, acting as an oligomer, inhibits enzymatic hydrolysis of triglycerides by preventing LPL dimerization [1]. ANGPTL4 has two common coding single nucleotid polymorphisms (cSNPs): E40K and T266M. The E40K substitution prevents Angptl4 oligomer formation, which leads to reduced Angptl4 mediated inhibition of LPL activity [2].

Studies in large Western and Asian population cohorts have proved an independent association between elevated triglyceride levels and CVD risk [3, 4].

ANGPTL4 concentration has a positive correlation with TG in patients with the metabolic syndrome [5]. A cohort study described a significant negative correlation between HDL-cholesterol and ANGPTL4 [5, 6]. Therefore, ANGPTL4 could be associated with disorders of lipid metabolism. This is also supported by human genetic data including genome wide association studies [7].

Population-based [8] studies and results in over 30,000 individuals from non-diabetic [7] have confirmed To date, that the E40K loss-of-function variant is associated with significantly lower triglyceride levels [7, 8]. In contrast, the ANGPTL4 T266M cSNP, which is more prevalent than E40K, has been shown to have a smaller effect on triglyceride levels in non-diabetic populations [8].

The purpose of this study is to determine whether the ANGPTL4 E40K and T266M polymorphisms are associated with triglyceride levels in well characterized patients with T2D, and with CVD risk factors, such as metabolic syndrome and obesity in type 2 diabetic Tunisian population.

## Methods

### Patients

A total of 220 type 2 diabetic patients, recruited through the Fattouma Bourguiba hospital (Monastir, Tunisia), were diagnosed by angiography. Subjects were defined with cardiovascular disease (CAD) when presenting a stenosis >50 % in at least one major coronary artery. Subjects were defined without coronary artery disease (No CAD) when presenting a stenosis <50 in at least one major coronary artery. Hypertension was diagnosed as a blood pressure of higher than 140/90 mmHg, which was measured according to guidelines [9] and/or the current use of anti-hypertensive drugs. Diabetic subjects were defined by a fasting plasma glucose >7.0 mmol/L, or by the use of anti-diabetic drugs [10]. Obese subjects were defined by a BMI >30.0 Kg/m2. Data on age, sex, smoking, and smoking history were collected from the participants’ medical records or by direct interviews. Fasting concentrations of TC, TG, LDL-C, and HDL-C were measured using standard methods [11]. The metabolic syndrome (MetS) was diagnosed when at least three of the following criteria applied: triglycerides ≥150 mg/dl (1.7 mmol/l), high density lipoprotein (HDL) cholesterol <40 mg/dl (1.0 mmol/l) in men and <50 mg/dl (1.3 mmol/l) in women, blood pressure ≥130/≥85 mm Hg, or fasting glucose ≥100 mg/dl (5.6 mmol/l). This study was approved by our hospital ethical committee chaired by Pr Fekri Abroug, the reported investigations have been carried out in accordance with the principles of the Declaration of Helsinki as revised in 2008. All participants were of Tunisian origin and gave their informed consent for this study.

### Biochemical analysis

Blood samples were taken for biochemical analysis following overnight fasting. Tch, triglyceride and HDL-c concentrations were determined at accredited clinical laboratories using routine clinical methods. LDL cholesterol concentrations were calculated using the Friedewald equation [12].

### DNA analysis

Genomic DNA was prepared from white blood cells using the salting-out method [13]. Genotypes for the variant 9p21.3 were determined by polymerase chain reaction (PCR) amplification with fluorescent-labeled primers.

### Genotyping

#### T266M polymorphism

This polymorphism was genotyped using the PCR-RFLP (restriction fragment length polymorphism) method. Primer sequences are: forward primer: 5’- TGC CTC ATG GAG TGG CCT CT-3’ and reverse primer : 5’-TGT CCT CGC CAC CCA GGT-3’. Amplification was performed in a total reaction volume of 20 μl each, containing 0.2 μM deoxynucleotide triphosphates, 1× Taq buffer, 3 mM MgCl2, 0.2 μM forward primer, 0.2 μM reverse primer and 1.0 U Taq polymerase. Thermocycling consisted of an initial denaturation step of 94 °C for 4 min followed by 35 cycles of 94 °C for 1mn s, 61 °C for 30 s, 72 °C for 40 s and a final extension cycle of 72 °C for 7 min. The amplified products consisting of a 165 bp fragment was electrophoretically separated on 2 % agarose gels. The PCR products were digested with restriction enzyme NlaIII and incubated at 37 °C during one night. The digested products consisting of: 156 + 9 bp for C allele and 94 + 62 + 9 bp for T allele were analyzed on 3 % agarose gel.

#### E40K polymorphism

Allele specific primers for the ancestral (G) and derived (A) alleles of rs1333049: G > A, were designed to selectively amplify the relevant target sequences [14]. The sequence of the forward primer for the G allele was: 5′-CTT TGC GTC CTG GGA CGA-3′ and for A allele the sequence was: 5′-GCT TTG CGT CCT GGG ACA-3′. The internal control primer sequence was: 5′-TGA TCC GAT TCT TTC CAG C-3′. For all the forward primers a common reverse primer was used, with the sequence: 5′-GGC TCT CCT GGC TTG GAA G-3′. Amplification of ancestral and derived alleles was performed separately in a total reaction volume of 20 μl each, containing 0.2 μM deoxynucleotide triphosphates, 1× Taq buffer, 3 mM MgCl2, 0.2 μM allele specific forward primer, 0.3 μM reverse primer, 0.1 μM internal control primer and 1.0 U Taq polymerase. Thermocycling consisted of an initial denaturation step of 94 °C for 4 min followed by 35 cycles of 94 °C for 1mn s, 61 °C for 30 s, 72 °C for 40 s and a final extension cycle of 72 °C for 7 min. The amplified products consisting of a 222 and 223 bp fragment for the allele specific primers (G and A respectively) and 508 bp for the internal control were electrophoretically separated on 2 % agarose gels.

DNA bands were visualized by UV trans-illumination, the image documented using the BioCap MW software (ver. 11.01, Vilber Lourmat, France) and the genotype data were then calculated.

### Statistical analyses

All statistical analysis was performed using version 11.0 of the Statistical Package for the Social Sciences: SPSS (SPSS Inc., Chicago, Illinois, USA). Continuous variables are presented as mean (95 % confidence intervals), and categorical data are summarized as frequencies or percentages. Normal distribution of continuous variables was evaluated with the Kolmolgorov-Smirnov test, and differences among groups were analyzed by one-way analysis of variance (ANOVA). For categorical variables, differences between groups were evaluated by the chi-square test. Odds ratios (ORs) of CAD for TT genotype on rs1044250 (T266M variant) and GA genotype on rs116843064 (E40K variant), and other risk factors, were estimated by multivariate logistic regression analyses. The significance of multiplicative interactions between TT genotype (T266M variant) and GA genotype (E40K variant) and covariates was determined by a Logistic regression model. The relationship between genotype and cardiovascular disease was evaluated with the Mann–Whitney U-test. A 2-sided probability level of ≤0.05 was considered significant.

## Results

### Baseline characteristics of the study population

Table 1 shows clinical features of control group (with No CAD) and CAD group. Compared to control group, patients of the CAD group have an increased percentage of obesity, MetS, hypertension, insulin and BMI, TG, and LDL-C levels (all *p* < 0.05). No significant differences in age, sex, smoking, fasting plasma glucose, HbA1c and HDL-C levels were found between the groups (all *p* > 0.05).

**Table 1:** Baseline characteristics of the study population

	Control group (No CAD) Mean(95 % CI)	CAD Mean(95 % CI)	p
N	117	103	-
Age (years)	63,94(62,1–65,78)	66,13(64,21–68,07)	0,10
Sex (%men)	48,7	52,4	0,62
Smoking (%)	26,5	44,7	0,27
Fasting plasma glucose (mmol/L)	11,48(10,59–12,36)	12(10,96–13,05)	0,44
HbA1c (mmol/L)	10,48(9,72–11,23)	11,01(10,11–11,91)	0,37
BMI (kg/m2)	29,91(29,14–34,67)	36,23(30,41–45,06)	0,02
Obesity (%)	42,7	61,2	0,02
Metabolic syndrome (%)	26,5	52,4	<0,001
SBP (mm/Hg)	144,78(138,94–150,63)	145,68(139,99–151,37)	0,82
DBP(mm/Hg)	78(74,71–81,29)	87,60(83,21–92)	0,001
Hypertension (%)	52,1	75,7	<0,001
TC (mmol/L)	4,8(4,6–5)	4,87(4,63–5,11)	0,63
TG (mmol/L)	1,71(1,51–1,91)	2,90(2,62–3,17)	<0,001
HDL-C (mmol/L)	1,33(1,24–1,41)	1,21(1,10–1,33)	0,10
LDL-C (mmol/L)	3,22(3–3,44)	4,40(4,08–4,71)	<0,001
Insulin (%)	40,2	77,7	<0,001

Data presented is mean (95 % confidence intervals)

### Association between rs116843064 polymorphism (E40K), biochemical measurements and Clinical features

Biochemical measurements and clinical features with respect to various genotypes are listed in Table 2.

**Table 2:** Association between rs116843064 (E40K) polymorphism, biochemical measurements and clinical features

	Control group Mean(95 % CI)			CAD Mean(95 % CI)		
	EE (GG)	EK (GA)	p	EE (GG)	EK (GA)	p
N	105	12	-	74	28	-
Age (years)	63,48(61,53–65,44)	67,92(61,97–73,87)	0,22	65,73(63,43–68,03)	67,1(63,24–70,98)	0,5
Sex (%men)	46,7	66,7	0,78	50	60,7	0,43
Fasting plasma glucose (mmol/L)	11,56(10,64–12,49)	10,74(7,20–14,28)	0,26	12,52(11,32–13,73)	10,48(8,34–12,61)	0,11
HbA1c (mmol/L)	10,55(9,76–11,34)	9,85(6,82–12,88)	0,22	11,49(10,42–12,56)	9,57(7,87–11,27)	0,08
BMI (kg/m2)	31,6(28,34–34,87)	29,71(28,93–30,49)	0,05	41,47(31,94–45)	31,16(30,15–32,17)	0,02
Obesity (%)	50	41,9	0,06	75,4	58,1	0,03
Metabolic syndrome (%)	26,7	25	0,12	47,3	67,9	<0,001
SBP (mm/Hg)	144,63(138,68–150,58)	146,17(119,19–173,14)	0,062	144,44(136,97–151,92)	148,14(140,8–155,48)	0,49
DBP(mm/Hg)	78,13(74,77–81,49)	76,83(61,87–91,8)	0,08	87,32(82,10–92,55)	87,89(78,99–96,8)	0,84
Hypertension (%)	51,4	58,3	0,64	71,6	85,7	0,12
TC (mmol/L)	4,79(4,58–5)	4,87(4,15–5,6)	0,27	4,73(4,44–5,03)	5,18(4,76–5,61)	0,06
TG (mmol/L)	1,72(1,51–1,94)	1,59(1,21–1,97)	0,01	1,9(1,59–2,2)	1,65(1,19–2,32)	<0,001
HDL-C (mmol/L)	1,32(1,23–1,41)	1,43(1,19–1,67)	0,07	1,21(1,06–1,35)	1,23(1,03–1,44)	0,08
LDL-C (mmol/L)	3,2(2,97–3,44)	3,36(2,69–4,03)	0,06	4,36(3,99–4,73)	4,34(3,78–5,09)	0,07
Insulin (%)	41	33,3	0,33	75,7	82,1	0,44

Data presented is mean (95 % confidence intervals)

There are no differences between the two genotypes in the control group (all *p* > 0.005) except the TG level, it’s lower in the heterozygote EK (*p* < 0.05). This genotype has a larger effect in the CAD group, showing lower BMI value, obesity and MetS percentages (all *p* < 0.05) and especially lower TG levels (*p* < 0.001), compared to EE homozygote. No differences were found in age, sex, insulin percentages, fasting plasma glucose, HbA1C, TC, HDL-C and LDL-C levels between the two genotypes of rs116843064 polymorphism.

### Association between rs1044250 polymorphism (T266M), biochemical measurements and Clinical features

As shown on Table 3, among the three genotypes, there are no differences in age, sex, hypertension, insulin percentages, fasting plasma glucose, HbA1C, TC, HDL-C and LDL-C values between the two groups (all *p* > 0.05). Compared to control group, there is a significant linear decrease in the BMI value, obesity and MetS percentages among the three genotypes in the CAD group, these values are significantly lower in MM genotype carriers. Concerning the TG level, MM genotype has a significant lowering effect in the control group (*p* = 0.04), but this effect is larger in CAD group (*p* = 0.01).

**Table 3:** Association between rs116843064 (T266M) polymorphism, biochemical measurements and clinical features

	No CAD				CAD			
	TT	TM	MM	p	TT	TM	MM	p
N	62	40	5	-	32	55	16	-
Age (years)	66,18(63,02–69,35)	63,54(62,11–65,92)	59,84(55–64,69)	0,06	67,14(64,11–70,18)	65,37(62,38–68,38)	65,62(60,12–71,13)	0,11
Sex (%men)	47,7	45,2	57,9	0,65	57,1	44,4	62,5	0,62
Fasting plasma glucose (mmol/L)	10,7(8,43–12,97)	11,56(10,13–12,99)	11,69(10,16–13,23)	0,74	11,31(8,91–13,71)	11,7(10–13,4)	12,53(10,86–14,2)	0,12
HbA1c (mmol/L)	10,18(8,08–12,27)	10,61(9,37–11,85)	11,01(9,68–12,34)	0,76	10,36(8,09–12,64)	10,73(9,4–12,07)	11,49(9,98–13)	0,76
BMI (kg/m2)	30,91(29,35–32,46)	29,62(28,59–30,64)	29,44(27,62–31,26)	0,27	42,32(40,83–43,80)	39,94(38,78–41,09)	32,03(30,68–33,37)	0,01
Obesity (%)	52,6	47,7	48,1	0,08	81,3	69	46,7	0,02
Metabolic syndrome (%)	26,2	27,3	26,8	0,21	62,5	51,9	41,94	0,01
SBP (mm/Hg)	149,33(137,59–161,08)	146,22(137,78–154–67)	137,05(121,5–152,6)	0,40	148,69(139,3–158,08)	143,62(134,51–152,74)	143,56(131,21–155;92)	0,16
DBP(mm/Hg)	82,59(76,56–88,64)	75,15(68,69–81,62)	75,13(69,34–80,94)	0,13	90,42(82,99–97,86)	83,97(77,06–90,9)	90,37(81,87–98,88)	0,17
Hypertension (%)	59,1	57,1	55,1	0,28	70	75	81,4	0,06
TC (mmol/L)	4,55(4,21–4,89)	4,87(4,58–5,16)	5,3(4,7–5,9)	0,44	4,71(4,18–5,24)	4,81(4,43–5,2)	4,98(4,59–5,38)	0,36
TG (mmol/L)	1,98(1,4–2,56)	1,75(1,41–2,09)	1,55(1,23–1,86)	0,04	2,53(2,47–3,19)	2,83(1,83–3,84)	3,23(2,53–3,45)	0,01
HDL-C (mmol/L)	1,3(1,06–1,54)	1,31(1,16–1,45)	1,41(1,28–1,55)	0,49	1,15(0,94–1,36)	1,19(0,99–1,40)	1,25(1,07–1,43)	0,68
LDL-C (mmol/L)	2,77(2,48–3,06)	3,31(2,94–3,68)	3,01(2,46–4,55)	0,31	3,25(3,07–1,43)	4,51(3,54–5,49)	4,74(4,24–5,24)	0,09
Insulin (%)	46,2	45,4	45,5	0,12	51,4	61,3	62,2	0,09

Data presented is mean (95 % confidence intervals)

### Comparison between E40K and T266M effect on TG levels

Tables 2 and 3 show that the association of E40K and T266M with triglyceride levels remained significant in the two groups (*p* = 0.01 and *p* = 0.04 respectively), but the association is more significant in the CAD group (*p* < 0.001and *p* = 0.01 respectively), and as shown E40K polymorphism has a larger effect in TG levels than T266M polymorphism.

### Multivariable analysis

A multiple logistic regression analysis was performed with CAD as the dependent variable (Table 4). Analysis showed that TT genotype of rs1044250 (T266M variant) showed a protective effect on CVD risk in CAD group patients (OR 1.92, 95 % CI 0.60–1.42, *p* =0.05) compared with control Group patients (OR 1.17, 95 % CI 0.70–1.66, *p* = 0.72). Likewise, GA genotype of rs116843064 (E40K variant): (OR 0.74, 95 % CI 0.54–1.65, p =0.01) for the CAD group compared with control Group patients (OR 1.12, 95 % CI 0.68–1.74, *p* = 0.074). So, the two genotypes of ANGPTL4 variants play a protective role against CVD in Tunisian type 2 diabetic patients.

**Table 4:** Multivariable logistic regression analysis

	Control group			CAD		
	OR	95 % CI	p	OR	95 % CI	p
T266M :						
rs1044250 (TT vs. non TT)	1,17	0,70–1,66	0,72	0,92	0,60–1,42	0,05
E40K :						
rs116843064 (GA vs. GG)	1,12	0,68–1,74	0,074	0,74	0,54–1,65	0,01

## Discussion

The principal findings of this study are a confirmation of an association of the E40K and T266M variants with lower TG levels and predicts cardiovascular disease risk in 220 Type 2 diabetic individuals. These results are partly in agreement with the findings of Talmud et al. [8] who examined 6 ANGPTL4 SNPs and reported that E40K was associated with increased CVD risk and T266M showed effects only under conditions of postprandial stress. But the functionality of these potential “loss-of-function” variants needs validation.

Formation of Angptl4 oligomers by disulphide bonds in the coiled-coil N-terminus [15] is required to inhibit LPL activity. The E40K substitution destabilizes the protein after secretion, preventing the extracellular accumulation of oligomers and abolishing the ability of the Angptl4 protein to inhibit LPL activity [2].

Variant rs1044250 (T266M) is located in the C-terminal region of ANGPTL4 and is conserved across human, rat, and mouse species, supporting a functional role [5, 16–18]. Thus, it may be assumed that rs1044250 may influence cardiovascular risk by decreasing ANGPTL4 activity in endothelial cells increasing angiogenesis and vascular permeability.

Smart-Haljko et al. [19] demonstrate for the first time that the ANGPTL4 E40K and T266M variants are associated with lower triglyceride levels in the setting of T2D. In addition, their findings demonstrate that ANGPTL4 genotype status does not alter triglyceride response to a lifestyle intervention in the Look AHEAD study. The T266M TG effect is consistent with the European Atherosclerosis Research Study II CHD offspring study which showed that MM homozygotes had enhanced triglyceride clearance following an oral fat tolerance test [8].

Our findings are confirmed by another study of Muendlein et al. [20], they showed a positive association of circulating ANGPTL4 levels as well as of ANGPTL4 SNPs with future cardiovascular risk in angiographically characterized coronary patients.

## Conclusion

ANGPTL4 variants are associated with, not only lower fasting triglyceride levels, but also a decreased cardiovascular risk in T2D Tunisian patients.

### Availability of data and materials

We do not wish to share our data, they are confidential to our research unit.

## Abbreviations

ANGPTL4: Angiopoietin-like protein 4
ANOVA: Analysis of variance
BMI: Body mass index
CAD: Coronary artery disease
cSNP: Coding single nucleotid polymorphisms
CVD: Cardiovascular disease
DNA: Deoxyribonucleic acid
HbA1c: Glycosylated hemoglobin A1c
HDL-C: HDL cholesterol
LDL-C: LDL cholesterol
LPL: Lipoprotein lipase
MetS: Metabolic syndrome
OR: Odds ratio
PCR: Polymerase chain reaction
SNP: Single nucleotide polymorphism
SPSS: Statistical package for the social sciences
T2D: Type 2 diabetes
TC: Total cholesterol
TG: Triglyceride
